# Alpha-synuclein fragments trigger distinct aggregation pathways

**DOI:** 10.1038/s41419-020-2285-7

**Published:** 2020-02-03

**Authors:** Tasnim Chakroun, Valentin Evsyukov, Niko-Petteri Nykänen, Matthias Höllerhage, Andreas Schmidt, Frits Kamp, Viktoria C. Ruf, Wolfgang Wurst, Thomas W. Rösler, Günter U. Höglinger

**Affiliations:** 1 Department of Translational Neurodegeneration, German Center for Neurodegenerative Diseases (DZNE), 81377 Munich, Germany; 2 Munich Cluster for Systems Neurology (SyNergy), University of Munich, 81377 Munich, Germany; 3 Department of Neurology, School of Medicine, Technical University of Munich, 81675 Munich, Germany; 4 Protein Analysis Unit (ZfP), Biomedical Center (BMC), University of Munich, 82152 Planegg, Germany; 5 Metabolic Biochemistry, Biomedical Center (BMC), University of Munich, 81733 Munich, Germany; 6 Center for Neuropathology and Prion Research, University of Munich, 81733 Munich, Germany; 7 Institute of Developmental Genetics, Helmholtz Center Munich, 85764 Munich, Germany; 8 Department of Neurology, Hannover Medical School, 30625 Hannover, Germany

**Keywords:** Parkinson's disease, Parkinson's disease

## Abstract

Aggregation of alpha-synuclein (αSyn) is a crucial event underlying the pathophysiology of synucleinopathies. The existence of various intracellular and extracellular αSyn species, including cleaved αSyn, complicates the quest for an appropriate therapeutic target. Hence, to develop efficient disease-modifying strategies, it is fundamental to achieve a deeper understanding of the relevant spreading and toxic αSyn species. Here, we describe comparative and proof-of-principle approaches to determine the involvement of αSyn fragments in intercellular spreading. We demonstrate that two different αSyn fragments (1–95 and 61–140) fulfill the criteria of spreading species. They efficiently instigate formation of proteinase-K-resistant aggregates from cell-endogenous full-length αSyn, and drive it into different aggregation pathways. The resulting aggregates induce cellular toxicity. Strikingly, these aggregates are only detectable by specific antibodies. Our results suggest that αSyn fragments might be relevant not only for spreading, but also for aggregation-fate determination and differential strain formation.

## Introduction

Synucleinopathies are a group of neurodegenerative diseases collectively characterized by intracellular protein inclusions containing alpha-synuclein (αSyn). αSyn is a predominantly presynaptic, intrinsically unfolded protein of 140 amino acids (aa)^1–3^. Its primary structure comprises three distinct domains: (i) an N-terminal domain (aa 1–60) that binds lipids and adopts alpha helical structures, (ii) a central domain known as non-amyloid component (NAC) (aa 61–95) involved in aggregation, and (iii) a C-terminal acidic tail (aa 96–140) accountable for most interactions with other proteins and small molecules^4^. Currently, the exact physiological functions of αSyn are still elusive. Nevertheless, it became clearer that αSyn has versatile functions, likely achieved by the distinct biophysical properties of its three domains^5–9^.

Intercellular transmission of αSyn was proposed as a mechanism for pathology propagation from one brain area to another^10,11^. Since aggregation of αSyn appears as a pivotal event in the pathogenesis of synucleinopathies, understanding the specific mechanisms that lead to accelerated aggregation is crucial. Interestingly, up to 15% of total αSyn in Lewy bodies (LBs) is truncated^4,12^. Moreover, truncation of αSyn seems to correlate with accelerated aggregation and pathology in cell and mouse models^13–15^. Remarkably, fragments are emerging as a potential common feature observed also for other neurodegeneration-related proteins, such as amyloid precursor protein and tau^16^.

We hypothesized that αSyn fragments might be implicated in intercellular spreading of pathology. Therefore, we focused on the four main events of the spreading process: (i) the release of αSyn species into the extracellular space, (ii) their uptake into naïve cells, (iii) their seeding capacity within the recipient cells and (iv) subsequent toxic effects.

Lund human mesencephalic (LUHMES) cells were used throughout the study. They differentiate into mature and well-characterized human dopaminergic neurons, which makes them highly relevant for αSyn pathology^17–19^. Herein, we report that αSyn fragments are found in the extracellular space of αSyn-overexpressing cells. Furthermore, we describe two fragments that were rapidly taken up by cells and seem to mediate the aggregation of endogenous full-length αSyn (FL-αSyn) into different pathways. The resulting aggregates were only detectable with specific antibodies, and ultimately induced toxicity.

## Results

### αSyn fragments are present in conditioned medium of αSyn-overexpressing neurons

**Fig. 1:**
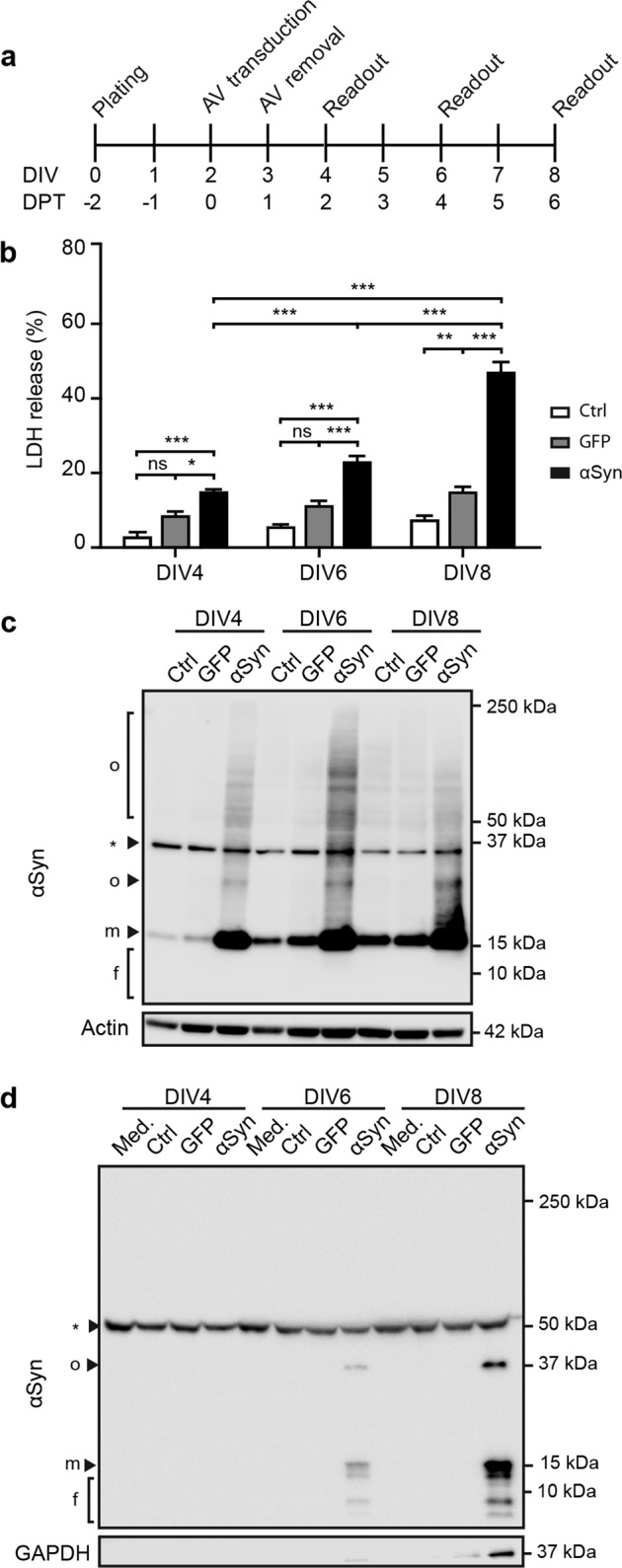
αSyn fragments are present in the conditioned medium of αSyn-overexpressing LUHMES neurons. **a** Experimental design. LUHMES cells, grown in differentiation medium from plating onward, were transduced with GFP or αSyn adenoviral vectors (AV) two days after plating. Viruses were removed 24 h after transduction. Cells and conditioned medium (CM) were harvested at the indicated readout times. DIV: days in vitro. DPT: days post-transduction. **b** αSyn-mediated toxicity. Cellular toxicity was monitored by lactate dehydrogenase (LDH) activity in the CM at the indicated readout times. Cells were either left untreated (Ctrl), challenged with a control AV expressing green fluorescent protein (GFP) or an AV expressing wild type αSyn. At DIV8, αSyn overexpression induced a considerably high toxicity, whilst GFP overexpression induced a mild toxicity, compared to untreated cells. LDH release values were related to a cell lysis positive control representing 100%. Data are presented as mean + standard error of the mean (SEM) from at least 3 biological repeats. ns: not significant, *p < 0.05, **p < 0.005, ***p < 0.001; two-way ANOVA with Tukey’s post hoc test. **c** αSyn-overexpression in cell homogenates. Cell lysates were analysed by Western blot at the indicated readout times. αSyn overexpression levels were stable between DIV4 and DIV8. Aggregation of αSyn is visible in overexpressing cells, but not in untreated control or GFP overexpression conditions. αSyn bands smaller than 15 kDa were not detected, indicating absence of fragmented αSyn. f: fragments; m: monomer; o: oligomer; *: unspecific band. Actin was used as loading control. **d** αSyn in CM. Western blot analysis of αSyn in CM at the indicated times reveals the presence of several αSyn species at DIV6 and DIV8. An oligomer band appears at 37 kDa (o), a monomer band at 15 kDa (m) and several fragmented αSyn bands ranging from 13 to 6 kDa (f). CM of untreated and GFP overexpressing cells did not contain detectable levels of αSyn. Unconditioned medium (Med.) was used as control for unspecific bands (*). GAPDH was used as control for cytoplasmic content in the CM.

To study the release of αSyn into the extracellular space, we used a previously reported αSyn overexpression model in differentiated LUHMES cells^19^. Cells were transduced with adenoviral (AV) vectors at day in vitro (DIV) 2 and toxicity, intracellular and extracellular αSyn were monitored over time (Fig. 1a). αSyn-mediated toxicity was assessed with lactate dehydrogenase (LDH) activity in culture medium (Fig. 1b). αSyn overexpression induced increased toxicity over time in comparison to GFP-overexpressing and untreated control cells, and reached approximately 50% at DIV8.

Next, we examined the intracellular αSyn species in whole cell homogenates (Fig. 1c). Expectedly, αSyn overexpression led to a stable increase in αSyn levels and marked intracellular aggregation. No intracellular αSyn fragments lower in molecular weight than FL-αSyn (15 kDa) were detectable at any time after transduction.

We then analyzed cell conditioned medium (CM) for extracellular αSyn species (Fig. 1d). Several forms of αSyn appeared in the CM at DIV6; an oligomeric species at approximatively 37 kDa, monomeric FL-αSyn at 15 kDa and several αSyn fragments ranging between 13 and 6 kDa. At DIV8, the level of all αSyn species increased considerably with concomitant increase of cytoplasmic leakage, pointing towards an accumulation of αSyn in the medium as a result of cell death.

### Multiple truncation events lead to extracellular αSyn fragments

**Fig. 2:**
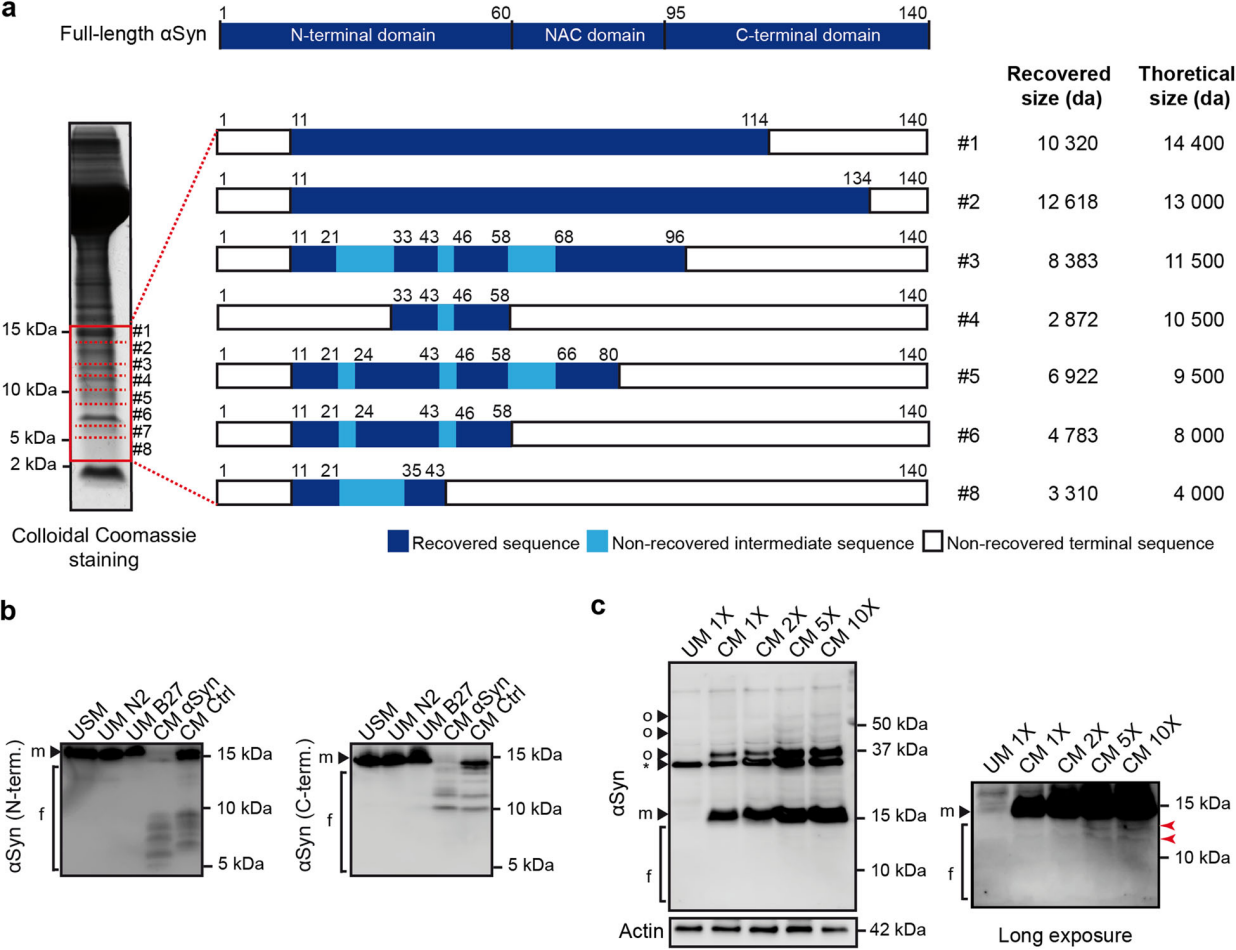
Identification of αSyn fragments in conditioned medium of αSyn-overexpressing LUHMES neurons and their uptake by naïve cells. **a** Schematic depiction of αSyn fragments detected in the CM of αSyn-overexpressing cells by LC-MS/MS. CM of αSyn-overexpressing cells was separated by gel electrophoresis. 8 sections, containing proteins between 3 and 15 kDa, were excised (red boxes) and analyzed by LC-MS/MS. The identified amino acid sequences of each section are represented. Recovered peptide sequences (dark blue boxes), not recovered sequences within recovered sequences (light blue boxes), and not recovered edge sequences (white boxes) are shown. Recovered and theoretical molecular sizes (corresponding to molecular size on the gel) of αSyn are displayed (right panel). In section 7, no signal corresponding to αSyn peptides was detected. **b** Generation of αSyn fragments in CM. Recombinant FL-αSyn was added to CM from αSyn-overexpressing cells (CM αSyn) or from untreated control cells (CM Ctrl). Unsupplemented medium (USM) and unconditioned medium supplemented with N2-supplement (UM N2) or B27-supplement (UM B27) were used as controls to ensure that the cleavage of αSyn is not caused by the culture medium or its supplements. Western blots with N-terminal (N-term.) and C-terminal (C-term.) antibodies show αSyn cleavage in CM of both untreated and αSyn-overexpressing cells, but not in unconditioned medium. f: fragments, m: monomer. **c** Uptake of αSyn fragments from CM into naïve αSyn knockout (KO) cells. αSyn KO cells (refer also to Supplementary Fig. S1) were used to eliminate the endogenous αSyn signal. Cells were treated with different concentrations of CM of αSyn-overexpressing cells for 6 h. Medium was either used unconcentrated (1X) or twice (2X), 5 times (5X) and 10 times (10X) concentrated. Unconditioned medium (UM) was used as negative control. Western blots indicate that monomeric αSyn (15 kDa) and an oligomeric species (37 kDa) are readily taken-up and are detectable with short exposure times (left panel), whereas uptake of αSyn fragments is only discernible from CM with higher concentrations (red arrows), using a longer exposure time. f: fragments, m: monomer, o: oligomer, *: unspecific bands.

Next, we aimed to confirm that the αSyn-immunoreactive bands smaller than 15 kDa in the extracellular space are indeed αSyn fragments, and to uncover their cleavage sites. Hence, we analyzed the CM of αSyn-overexpressing cells at DIV8 by liquid chromatography tandem mass spectrometry (LC-MS/MS) (Fig. 2a). Peptides corresponding to αSyn were found in all investigated gel sections, except gel section #7. One N-terminal and multiple C-terminal truncation events were detected (Fig. 2a). LC-MS/MS analysis confirmed the bands below 15 kDa observed in CM (Fig. 1d) as several αSyn fragments (Fig. 2a), and suggest that they result from proteolytic activity at multiple sites of αSyn. However, it was not possible to identify precise cleavage sites which would have allowed to delineate the protease(s) responsible for this cleavage pattern in our model.

### αSyn fragments are generated extracellularly and further taken up by naïve cells

Since αSyn fragments were observed exclusively in the extracellular space (Fig. 1c, d), we investigated whether they are generated extracellularly. Incubation of recombinant FL-αSyn with unconcentrated conditioned medium, followed by WB analysis with N- and C-terminal αSyn antibodies revealed that cleavage of recombinant αSyn occurred in CM of both αSyn-overexpressing and untransduced control cells, whereas the medium alone and the tested supplements did not lead to the cleavage of αSyn. In this experimental design, CM-derived FL-αSyn and fragments were below the detection limit. Therefore, only added recombinant FL-αSyn and its cleavage products could be visualized (Fig. 2b). The presence of cleaved αSyn in untransduced controls indicates that αSyn is very likely cleaved in the extracellular space by a protease that is secreted by cells under physiological conditions.

We next asked whether the fragments generated in the extracellular space of αSyn-overexpressing cells are taken up by naïve cells. To eliminate endogenous αSyn signal, we used αSyn knockout (KO) LUHMES neurons as recipient cells (Fig. 2c). An αSyn KO LUHMES cell line was generated by CRISPR/Cas9 genome editing and showed absence of αSyn throughout differentiation (Supplementary Fig. S1). For uptake evaluation, naïve αSyn KO cells were incubated with several concentrations of CM of wild-type (WT) αSyn-overexpressing cells. A dose-dependent uptake of monomeric FL-αSyn and a 37 kDa oligomer was observed, but no fragments could be detected upon short exposure time (Fig. 2c). With a longer exposure time, however, a signal corresponding to an uptake of αSyn fragments was visible with the highest concentrations of CM (5X and 10X). This finding provides evidence that αSyn fragments can be taken-up by naïve cells. Since in these experimental conditions the uptake of FL and oligomeric αSyn species prevailed over fragmented αSyn, we conducted further experiments with recombinant αSyn fragments.

### αSyn fragments differentially influence the aggregation of full-length αSyn

**Fig. 3:**
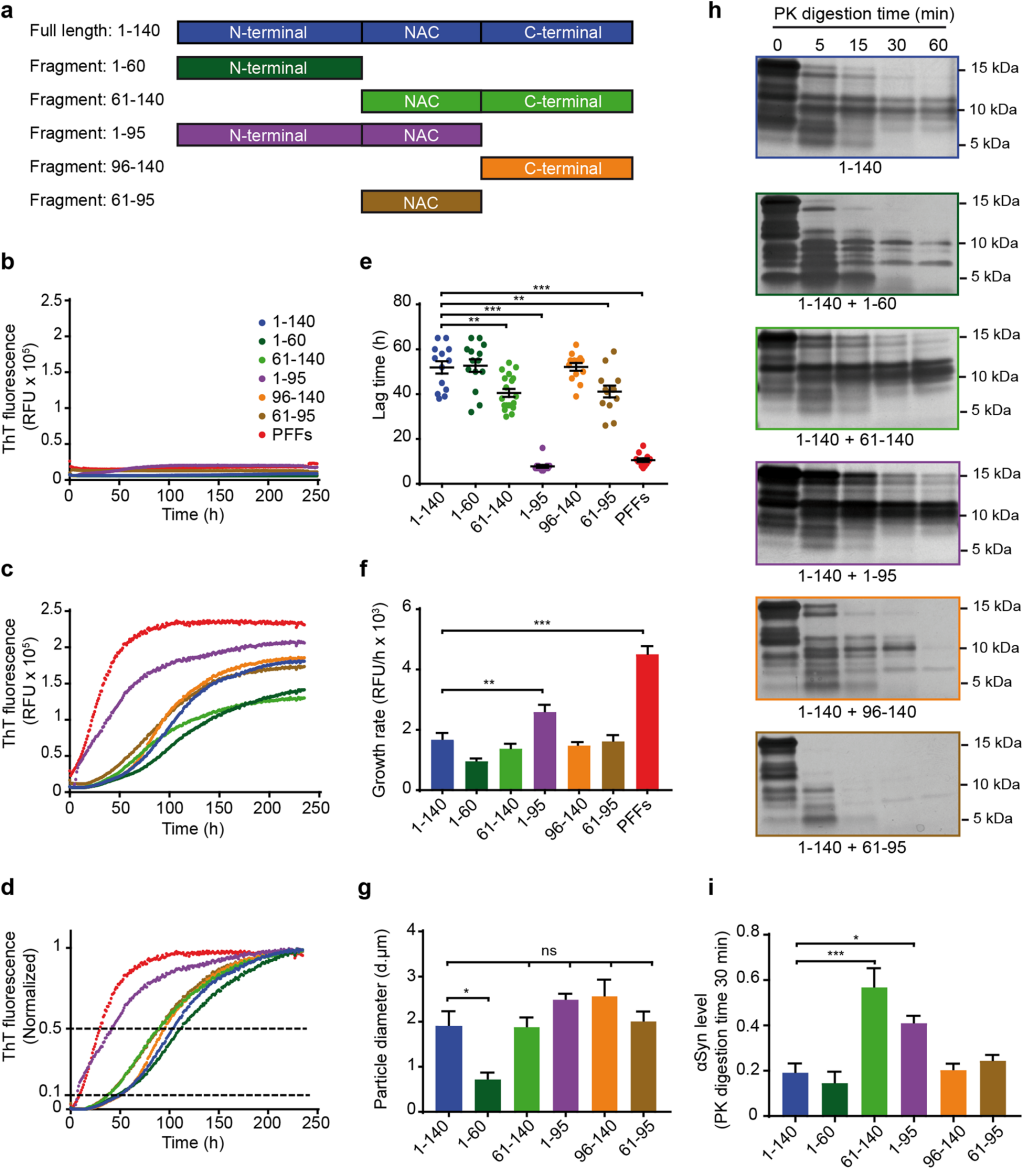
Recombinant αSyn fragments show differential effects on aggregation of full-length αSyn in a cell-free aggregation assay. **a** Schematic overview of recombinant FL-αSyn and fragments used in the study: Full length (1–140), N-terminal fragment (1–60), NAC + C-terminal fragment (61–140), N-terminal + NAC fragment (1–95), C-terminal fragment (96–140) and NAC fragment (61–95). The same names and color-code are used in all the panels. **b**–**d** Seeding effect of recombinant αSyn fragments on recombinant FL-αSyn in a cell-free aggregation assay. For the aggregation reactions, different fragments described in **a** were used as seeds and FL-αSyn was used as a substrate at a molar ratio of 1:10, respectively. Aggregation kinetics was monitored by measurement of thioflavin T (ThT) signal. αSyn pre-formed fibrils (PFFs) were used as positive control for seeding. RFU: relative fluorescence unit. Aggregation curves of αSyn fragments without addition of FL-αSyn substrate are shown in **b**. Aggregation curves with addition of FL-αSyn substrate as raw fluorescence data are shown in **c**, and normalized fluorescence ratio data from **c** are shown in **d**. The lower (0.1) and the upper (0.5) fluorescence thresholds of the total signal are shown with dotted lines. Aggregation curves are presented as mean from at least 3 repeats with 3 technical replicates each. **e** Lag time of aggregation seeded with different αSyn fragments. Lag times are presented as the time at which each curve reached 10% of the total fluorescence signal. Lag times were strikingly reduced in aggregation reactions seeded with PFFs and fragment 1–95. Each dot of the scatter plot represents one aggregation curve. Data are presented as mean ± SEM from at least 3 repeats with 3 technical replicates each (black lines). **p < 0.005, ***p < 0.001; one-way ANOVA with Tukey’s post hoc test. **f** Growth rate of aggregation seeded with different αSyn fragments. Aggregation growth rate is presented as a measure of increased ThT fluorescence per hour. Growth rates were significantly higher in aggregation seeded with the fragment 1–95 and the PFFs (positive control), and remained unchanged in all other aggregation reactions. Data are presented as mean + SEM from at least 3 repeats with 3 technical replicates each. **p < 0.005, ***p < 0.001; one-way ANOVA with Tukey’s post hoc test. **g** Particle size of aggregates seeded with different αSyn fragments. Particle size of aggregates was measured with dynamic light scattering (DLS) after the aggregation reactions were finished. Except for fragment 1–60, which resulted in significantly smaller fibrils, no significant differences in fibril size were observed across the rest of the samples. Data are presented as mean + SEM of particle diameter (d.nm) from at least 3 repeats. *p < 0.05; one-way ANOVA with Tukey’s post hoc test. **h**, **i** Proteinase K (PK) resistance of aggregates produced by seeded aggregation with different αSyn fragments. Aggregation was carried out with the same molar ratio of seed to substrate (1:10) as described above. PK digestion was performed at 37 °C for the indicated amounts of time. Protein separation by gel electrophoresis followed by silver staining revealed that aggregates seeded with fragments 61–140 and 1–95 were more PK resistant than the other samples. **i** A normalized optical density quantification of αSyn digestion at 30 min. Data are presented as mean + SEM from at least 3 repeats. *p < 0.05, ***p < 0.001; one-way ANOVA with Tukey’s post hoc test.

To characterize the effects of αSyn fragments on FL-αSyn aggregation by a systematic approach, distinct recombinant fragments containing one or two adjacent full domains of αSyn were chosen (Fig. 3a). Noteworthy, the fragments identified in sections #3 and #6 in the CM of αSyn-overexpressing cells (Fig. 2a) were very similar to recombinant fragments 1–95 and 1–60.

In a cell-free aggregation assay, non-aggregated recombinant fragments were used as seeds and monomeric FL-αSyn was used as a substrate with a molar ratio of seed to substrate of 1 to 10 in all the reactions. αSyn preformed fibrils (PFFs) represent a positive control for seeded aggregation due to their well-established capacity as highly efficient seeds^20,21^ and FL-αSyn represents the baseline reference for aggregation kinetics.

The incubation of seeds without substrate showed that they do not have a considerable input on the aggregation curves (Fig. 3b). When the substrate was added to the reactions, the ThT signal increased substantially indicating that it corresponds primarily to the aggregation of FL-αSyn (Fig. 3c). Generally, the obtained aggregation curves had a characteristic sigmoidal amyloid aggregation shape (Fig. 3c). The apparent lag time was reported as the time at which ThT signal reached 10% of its total increase, and growth rates were calculated as the difference in RFU per hour between 10 and 50% of the total ThT signal increase (Fig. 3d).

As expected, PFFs dramatically accelerated the aggregation. Strikingly, fragment 1–95 behaved similarly and reduced the apparent lag time to the same extent as PFFs. Fragments 61–140 and 61–95 also accelerated the aggregation but more moderately. The growth rate of seeded aggregation with PFFs was higher than the one seeded with fragment 1–95 implying that even though both initiated aggregation with a very similar efficiency, the aggregation seeded with fragment 1–95 required a longer time to reach a plateau as compared to PFFs. Similarly, fragments 61–140 and 61–95 reduced the lag time without detectable effect on the growth rate in comparison with monomeric FL-αSyn. Fragments 1–60 and 96–140 had no detectable effect on aggregation kinetics (Fig. 3e, f).

Next, we studied whether seeding with different αSyn fragments would result in aggregates with different properties. Dynamic light scattering was used to measure the particle diameter of the different aggregates. No significant differences in particle size between the different aggregates were observed, except for the ones seeded with fragment 1–60 (Fig. 3g). Interestingly, both fragments 61–140 and 1–95 resulted in aggregates with a higher PK resistance (Fig. 3h, i). PFFs were not used as seeds in these latter experiments, since the aggregates formed by FL-αSyn are identical to PFFs.

Taken together, these data showed that the NAC domain is essential for aggregation, and non-aggregated αSyn fragments that contain the NAC domain flanked by either the N- or the C-terminal domain can act as effective seeds that differentially influence the aggregation of FL-αSyn.

### αSyn fragments are taken up by LUHMES neurons

**Fig. 4:**
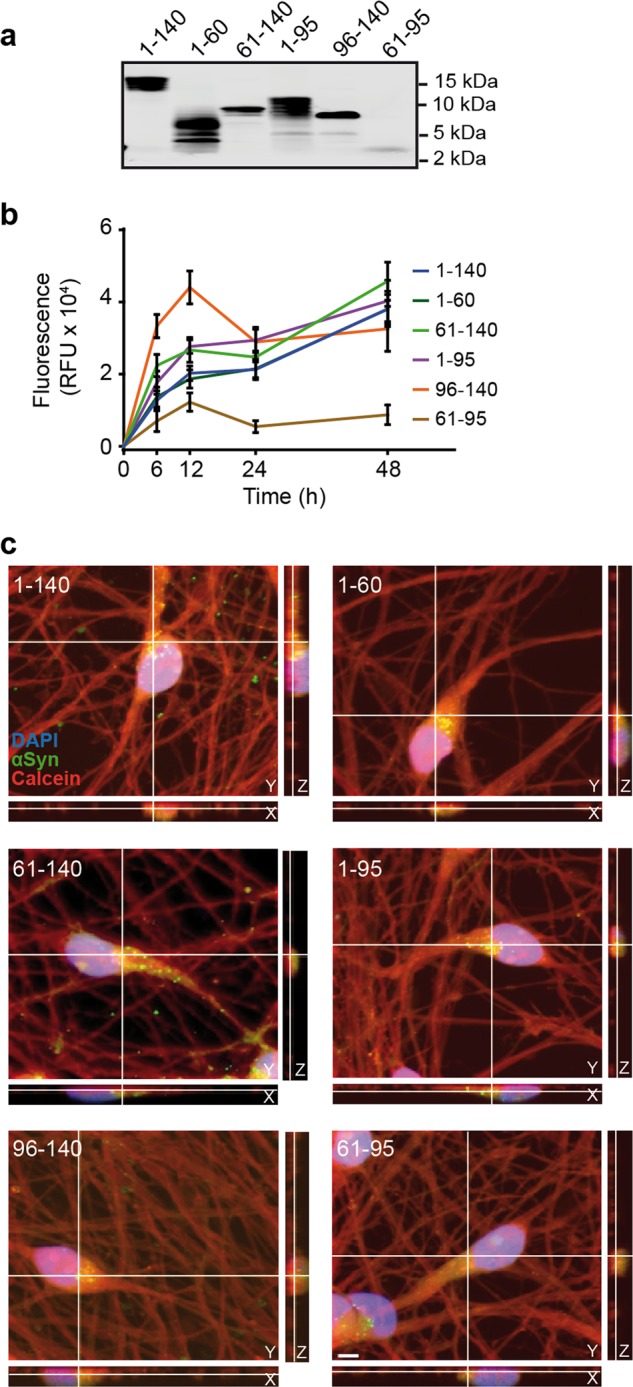
Recombinant αSyn fragments are readily taken-up by naïve LUHMES neurons. **a** Fluorescent gel imaging of recombinant ATTO-488-labeled FL-αSyn and fragments (depicted in Fig. 3a). All labeled αSyn species have a fluorescent signal at the expected molecular size. **b** Uptake kinetics of recombinant FL-αSyn and fragments. Cells were treated with labeled αSyn at DIV4. At the indicated times, intracellular fluorescence was measured. Extracellular fluorescence was quenched using trypan blue. RFU: relative fluorescence unit. **c** Uptake of ATTO-488-labeled αSyn fragments 48 h after treatment. Confocal images show orthogonal projections of Z-stacks with a clear intracellular localization of recombinant FL-αSyn and fragments. A calcein red-orange filling (red), DAPI staining (blue) and labeled recombinant αSyn (green) are shown. Scale bar: 5 µm.

To investigate the relevance of recombinant αSyn fragments in intercellular spreading, we addressed their uptake into human dopaminergic neurons. Recombinant FL-αSyn and fragments were fluorescently labeled with ATTO-488 and successful labeling was verified with gel electrophoretic separation followed by fluorescence imaging (Fig. 4a).

Cells were treated at DIV4 for the indicated amounts of time and intracellular fluorescence was measured (Fig. 4b). Uptake was rapid during the first 12 h, followed by a slower phase between 12 and 48 h. The uptake kinetics of all labeled species was similar except for fragment 61–95, i.e. the NAC domain, which appears to be taken up less efficiently most likely because of its highly hydrophobic nature.

Uptake at 48 h after treatment was further ascertained with confocal imaging (Fig. 4c). The fluorescent signal corresponding to the different labeled αSyn species was contained within the cells, thus showing their intracellular localization.

### Fragments 1–95 and 61–140 induce different aggregation patterns

**Fig. 5:**
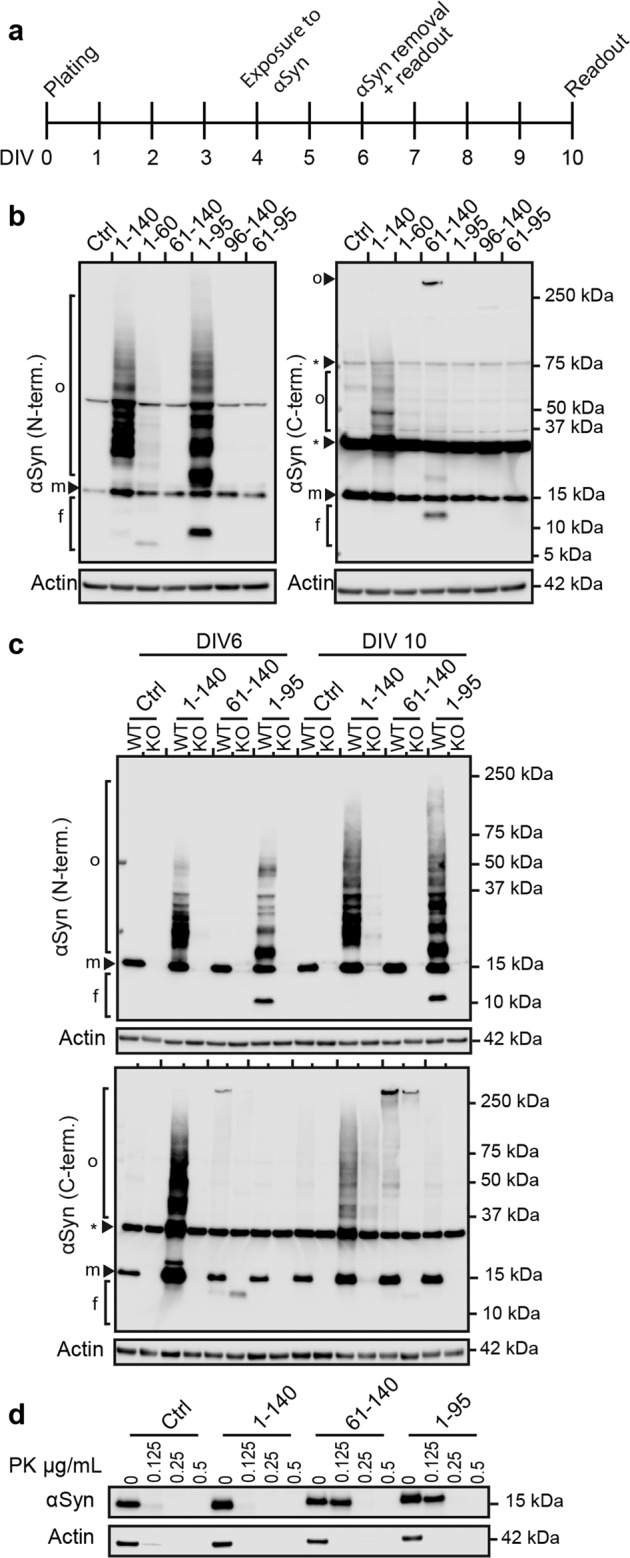
αSyn fragments induce different aggregation patterns in LUHMES neurons. **a** Experimental design. Cells were differentiated for 4 days before treatment. Treatments with recombinant FL-αSyn and fragments were carried out for 48 h and then removed. Two readout time points DIV6 and DIV10, were chosen. **b** Intracellular seeding potency of recombinant FL-αSyn and fragments at DIV6. Western blot analysis was performed with antibodies targeting either the N-terminal domain (N-tem.) or the C-terminal domain (C-term.) of αSyn. Fragment 1–95 induced intracellular aggregation which could only be visualized by an N-terminal αSyn antibody, whereas fragment 61–140 induced intracellular aggregation which only could be visualized by a C-terminal αSyn antibody. f: fragments, m: monomer, o: oligomer, *: unspecific bands. Actin was used as loading control. **c** Intracellular seeding potency of recombinant FL-αSyn and fragments in wild-type (WT) and αSyn knockout (KO) LUHMES cells. Western blots with N- and C-terminal antibodies in WT cells at DIV6 reveal the same aggregation patterns as in **b**, whereas no aggregation was detectable in the αSyn knockout cells. In WT cells at DIV10, the aggregation process seems continue after removal of treatment. f: fragments, m: monomer, o: oligomer, *: unspecific bands. Actin was used as loading control. **d** Proteinase K (PK) resistance of intracellular aggregates produced by seeding with recombinant FL-αSyn and fragments. PK was added with the indicated concentrations to cell lysates from DIV10 treated cells, and incubated at 37 °C for 30 min. Intracellular aggregates induced by fragments 61–140 and 1–95 show a higher PK resistance compared to aggregates formed by recombinant FL-αSyn (1–140) and untreated controls (Ctrl). Actin was used as a control for both sample loading and PK digestion efficiency.

The relevance of αSyn species in spreading depends on their capacity to seed intracellular aggregation after uptake. Therefore, we investigated whether the recombinant αSyn fragments have seeding potential on cell-endogenous FL-αSyn. Since the physiological neuronal concentration of αSyn was previously estimated to be at least 30 µM^22,23^, we treated differentiated LUHMES neurons with 3 µM recombinant FL-αSyn and fragments to maintain the 1:10 seed to substrate ratio. Intracellular αSyn was examined using antibodies targeting the N- or C-terminus of αSyn. We chose two readout times to analyze the immediate effects (DIV6) and the persistent effects after removal of treatments (DIV10) (Fig. 5a).

At DIV6, intracellular aggregation was only observed in cells exposed to FL-αSyn and fragments 61–140 and 1–95 (Fig. 5b). Interestingly, the treatments resulted in different aggregation patterns, implying different aggregation pathways. Also, fragment 1–60 seemed to trigger a less prominent, distinct aggregation pathway. Fragments 96–140 and 61–95 did not induce detectable intracellular aggregation, which may be due to the lack of the aggregation-prone NAC domain in the former, and a highly insoluble state and inefficient uptake for the latter (Fig. 4b, c).

Importantly, while intracellular aggregation ensuing from FL-αSyn treatment was detected by both N- and C-terminal antibodies, aggregates resulting from treatment with fragments 1–95 and 61–140 were only detectable by the N-terminal or the C-terminal antibody, respectively. This intriguing finding could indicate that endogenous αSyn is recruited for aggregation, but the generated aggregates only expose seed-specific epitopes. Alternatively, intracellular aggregation observed at DIV6 potentially resulted from self-aggregation of the different αSyn treatments.

### Endogenous αSyn is required for intracellular fragment-seeded aggregation

To clarify whether endogenous αSyn is recruited for aggregation seeded by fragments 61–140 and 1–95, we compared the intracellular seeding activity in WT and αSyn KO cells. At DIV6, the characteristic aggregation patterns were present in WT cells, whereas they either completely failed to appear for FL-αSyn and fragment 1–95, or were substantially reduced for fragment 61–140 in αSyn KO cells (Fig. 5c). This indicates that endogenous αSyn is not merely recruited for fragment-seeded aggregation, but is essential for it to occur. Notably, the presence or absence of endogenous αSyn seems to influence either the uptake or the intracellular processing of recombinant αSyn fragments. In WT cells, FL-αSyn and fragments 61–140 and 1–95 were clearly visible at the expected molecular size with short exposure times. In αSyn KO cells, longer exposure times had to be applied in order to visualize them (Supplementary Fig. S2).

Interestingly, aggregation seeded with FL-αSyn and fragments 61–140 and 1–95 continued for at least four days after treatment removal in WT cells (Fig. 5c). This emphasizes that endogenous αSyn is seemingly the fundamental substrate for maintaining the aggregation process. Moreover, these aggregates exhibited a clear shift of αSyn from soluble to insoluble state between DIV6 and DIV10 (Supplementary Fig. S3).

Furthermore, in accordance with our findings under cell-free conditions (Fig. 3h), intracellular aggregates seeded by fragments 61–140 and 1–95 showed greater PK resistance than aggregates seeded with FL-αSyn (Fig. 5d).

### Fragments 61–140 and 1–95 induce toxicity

**Fig. 6:**
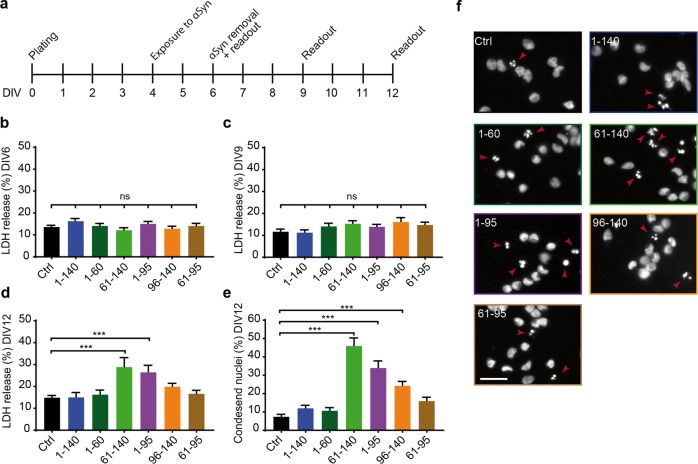
αSyn fragments that induce intracellular aggregation also induce toxicity. **a** Experimental design. Cells were differentiated for 4 days before start of treatment. Treatments with recombinant FL-αSyn and fragments were carried out for 48 h. Toxicity was assessed at three readout times. **b**–**d** Toxicity measured by LDH release in cells treated with recombinant FL-αSyn and fragments at DIV6 (**b**), DIV9 (**c**) and DIV12 (**d**). At DIV12, recombinant αSyn fragments 61–140 and 1–95 induced toxicity levels of approximately 30% compared to a cell lysis positive control. Data are presented as mean + SEM from 4 biological repeats with at least 4 technical replicates each. ns: not significant, ***p < 0.001; one-way ANOVA with Tukey’s post hoc test. **e, f** Percentage of condensed nuclei in cells treated with recombinant FL-αSyn and fragments at DIV12. Cells were fixed and nuclei were stained with DAPI. Recombinant αSyn fragments 61–140 and 1–95 induced higher toxicity levels in accordance with LDH data (**d**). Data are presented as mean + SEM from 3 biological replicates with at least 3 technical repeats each and 3 pictures per technical repeat. ***p < 0.001; one-way ANOVA with Tukey’s post hoc test. **f** Representative pictures of DAPI stained nuclei from cells treated with recombinant FL-αSyn and fragments at DIV12. Red arrowheads indicate condensed nuclei. Scale bar: 30 µm.

To investigate the relevance of αSyn fragments for pathology, we examined whether these specific fragments induced toxicity subsequent to intracellular aggregation. Therefore, we monitored the cellular toxicity by measuring LDH activity in the CM over time (Fig. 6a). None of the treatments showed an immediate toxic effect at DIV6 and DIV9 (Fig. 6b, c). However, only fragments 61–140 and 1–95 induced toxicity that first appeared at DIV12 (Fig. 6d). To confirm this observation, cells were fixed and stained with 4′,6-diamidino-2-phenylindole (DAPI) at DIV12 to quantify the percentage of condensed nuclei representing the final stage of apoptosis (Fig. 6e, f). This method also showed an increased percentage of condensed nuclei in cells treated with fragments 61–140 and 1–95 in comparison to those treated with FL-αSyn and the untreated control.

## Discussion

The specific role of different αSyn fragments in the formation of pathological inclusions remains elusive. Our study demonstrates that αSyn-mediated pathology leads to the appearance of extracellular αSyn fragments. Moreover, recombinant fragments 1–95 and 61–140 induce very distinct aggregation dynamics of FL-αSyn, result in aggregates with increased PK-resistance, and induce neurotoxicity. Intriguingly, aggregates seeded by these fragments are only recognized by distinct antibodies. Since fragments, in distinction to fibrils, are highly soluble and diffusible, they might be very relevant intercellular spreading species in human diseases and might thus be attractive therapeutic targets.

Isolating extracellularly generated αSyn fragments is technically challenging as their amounts are extremely low. Moreover, since the specific protease(s) responsible for the different fragments detected by LC-MS/MS were not clearly identified (Fig. 2a), and due to the margin of error inherent to peptide sequencing, the exact sequence of these fragments was not unambiguously ascertained. Thus for further analysis, we used a set of clearly defined recombinant αSyn fragments. Although, the sequences of these recombinant fragments differ from the ones detected by LC-MS/MS, they presented two major advantages. First, they are clearly defined species which are available in required amounts for the study. Secondly, they alternatively combine all the main domains of αSyn, thus allowing the evaluation of the effects of the full domains, rather than the partially-cleaved domains as seen in the fragments detected by LC-MS/MS. Noteworthy, recombinant fragment 1–95 mimics most closely the c-terminally cleaved fragment 11–96 identified in the CM of αSyn-overexpressing cells. This fragment indeed showed striking effects. The other recombinant fragments used in our experiments did not resemble those observed in the supernatant, and may thus be considered as control peptides. Although their sequences may not be considered ‘biologically accurate’, the evaluation of the effect of distinct domains and domain combinations of αSyn revealed interesting insights in regards to their role in aggregation and spreading.

Several cleaved forms of αSyn were previously found in healthy and diseased human brain samples^24^, as well as in biological fluids such as cerebrospinal fluid, blood^25–27^, and saliva^28^. However, whether their presence in biological fluids and brain samples play a role in spreading of pathology in humans is not clearly established thus far. Notably, fragments with the sequences 1–96 and 65–140, which highly correspond to fragments 1–95 and 61–140 used in our study, were identified in human brains^29,30^. To our knowledge, our findings provide first experimental evidence that these two specific αSyn fragments have distinct seeding properties and may be involved in further propagation of αSyn pathology.

Interestingly, recombinant fragments 1–95 and 61–140 were found to induce toxicity in primary microglia and increase the release of inflammatory cytokines^31^, further underlining their biological relevance since they might play synergetic pathological roles in both neurons and glial cells.

Our results suggest that fragments are generated by extracellular proteolytic cleavage of αSyn. Several extracellular proteases are known to cleave αSyn, e.g. plasmin^32^, neurosin^33,34^ and several metalloproteinases^35–38^. Although we were not able to identify the active protease(s) in our experimental system, a comparison between our MS data and previously published cleavage sites of αSyn suggests that plasmin and matrix metalloproteases 3 and 9 could be suitable candidates^32,36^. Further investigation is needed upon the identification of these proteases. Overall, incomplete proteolytic cleavage of extracellular αSyn seems to exacerbate αSyn pathology^35,36^. Here, we provide evidence that αSyn lacking either the N- or the C-terminus leads to neurotoxicity. Hence, it is necessary to carefully approach the modulation of proteolytic activity as a therapeutic strategy. Regarding our findings, it might be most beneficial to inhibit proteolysis that occurs at the N- and C-termini and promote more specifically cleavage of the NAC domain of αSyn.

While the aggregation-prone characteristic of C-terminally truncated αSyn is well established^39–43^, little is known about the aggregation activity of N-terminally truncated αSyn. In this study, non-aggregated fragments containing distinct αSyn domains and domain combinations were used to seed the aggregation of FL-αSyn with a molar ratio similar to the one found in LBs^12,44,45^. We show that non-aggregated, NAC domain containing αSyn fragments can act as powerful seeds. They increase the PK-resistance of aggregates, despite of distinct aggregation dynamics. Furthermore, recombinant fragments 1–95 and 61–140 induce very distinct intracellular aggregation patterns, emphasizing that various fragments lead to different aggregates thereby evoking the novel hypothesis of fragments as fate determining factors for aggregation.

Remarkably, endogenous αSyn was essential for aggregation initiation and progression, which was also recently shown in vivo^46^. Importantly, intracellular aggregates derived from seeding with fragments 1–95 and 61–140 also induced a higher cellular toxicity than the ones seeded with FL-αSyn. While it is difficult to conclude with certainty that toxicity derives directly from the different types of intracellular aggregates, the observation that toxicity had a delayed onset and only appeared where PK-resistant aggregates accumulate overtime indicates that the two events might be related. Together, these findings suggest that seeding with different fragments can potentially lead to the generation of various αSyn strains.

αSyn strains are emerging as a potential explanation for why different brain areas and cell types are affected between various synucleinopathies^47^. Presently, the direct implication of αSyn fragments in the differential formation of different strains has never been adressed. Nonetheless, αSyn fragments were found to have variable solubilities in different synucleinopathies, which directly implicates their seeding activities. Indeed, αSyn fragments seem to be more soluble in multiple system atrophy (MSA) than in Parkinson’s disease (PD) and dementia with Lewy bodies (DLB) brain samples^48^. Moreover, a comparison across several studies reveals slight differences in αSyn fragment patterns between PD, DLB, and MSA. In PD, larger fragments (~12–13 kDa) seem to be enriched, whereas smaller fragments (~6–8 kDa) appear to be more abundant in DLB. Detectable but less prominent accumulation of αSyn fragments was also detected in MSA^48,49^. Accordingly, the question arises whether differential proteolytic activities might be a driving mechanism that can determine the pathogenesis of different synucleinopathies. More recently, fibrils prepared with homogenous N- or C-terminally truncated αSyn were shown to exhibit distinct prion-like cross-seeding activities in mice^50^. Together with our findings, this strongly implicates the contribution of αSyn fragments in the formation of several strains of aggregates. In relation to our data, further characterization of structural and conformational changes, and the ability to maintain the same characteristics throughout several cycles of aggregation, would provide a deeper understanding of whether αSyn fragments effectively lead to formation of different strains.

Intriguingly, aggregates seeded with fragments 1–95 and 61–140 only showed immunoreactivity toward antibodies that target their corresponding seeding species. Thus, the unrecognized epitopes are either hidden or missing. Accordingly, different conformations of FL-αSyn could be formed so that only epitopes of the added seeds are exposed during the aggregation. Alternatively, fragments might trigger the conversion of endogenous FL-αSyn into fragmented forms containing only the epitope of the seed. A similar scenario has been observed with fibrils produced with C-terminally truncated αSyn that activated caspase 1, which in turn converted endogenous FL-αSyn into the same C-terminally truncated form^42^.

Notwithstanding, these findings might have implications for immunotherapeutic approaches, which are currently considered as promising disease-modifying strategies for synucleinopathies^51^. To produce therapeutic antibodies that efficiently target extracellular αSyn and prevents it from further spreading, it is crucial to thoroughly characterize spreading αSyn species. Here, we describe different intracellular aggregates that can be completely ‘invisible’ to certain antibodies. Consequently, a comprehensive screening for a variety of epitopes on several forms of patient-derived αSyn could achieve a better estimation on specific species to target. Moreover, it may be necessary to consider disease-, or even patient-specific mixtures of antibodies.

In summary, we explored the potential of distinct αSyn fragments as spreading species of αSyn pathology across neurons. Our findings provide novel experimental evidence of the direct involvement of various forms of truncated αSyn in differential seeding activities, since they were able to drive aggregation into distinct pathways and induce latent neurotoxicity. These multiple characteristics of αSyn fragments strongly emphasize their relevance as promising therapeutic targets for synucleinopathies.

## Methods and materials

### Cell culture

Undifferentiated Lund human mesencephalic (LUHMES) cells^17^ were expanded on 50 µg/mL poly-l-ornithine (Sigma-Aldrich, St. Louis, MO) coated T75 flasks (EasYFlasks, Nunclon DELTA, VWR, Darmstadt, Germany) in DMEM/F12 (Sigma-Aldrich) supplemented with 1% N2 supplement (Life Technologies, Carlsbad, CA) and 0.04 µg/mL basic fibroblast growth factor (bFGF; PeproTech, Rocky Hill, CT).

For experiments, cells were seeded on either T25 flasks or multi-well-plates (Nunc MicroWell plates, Thermo Fisher Scientific, Waltham, MA) sequentially coated with 50 µg/mL poly-l-ornithine (Sigma-Aldrich) and 5 µg/mL bovine fibronectin (Sigma-Aldrich). To induce differentiation of LUHMES cells into dopaminergic neurons, differentiation medium consisting of DMEM/F12 with 1% N2 supplement, 1 µg/mL tetracycline, 0.49 µg/mL dibutyryl cyclic-AMP (Sigma-Aldrich) and 2 ng/mL glial cell-derived neurotrophic factor (GDNF; R&D Systems, Minneapolis, MN) was used. Cell density was kept at 100,000 cells/cm^2^ across all flasks and well plate formats. Cells were kept at all times in standard cell culture conditions at 37 °C, 5% CO_2_, and water-saturated air. Cells were routinely tested for mycoplasma contamination.

### Transduction with adenoviral vectors

Adenoviral vectors expressing human wild-type alpha synuclein (αSyn) or green fluorescent protein (GFP) under a cytomegalovirus promoter (BioFocus DPI, Leiden, Netherlands) were added to LUHMES cells 48 h after start of differentiation with a multiplicity of infection (MOI) of 2, as previously described^52,53^. Untreated controls were supplemented with fresh differentiated medium without adenoviral vectors. After 24 h, the adenoviral vectors were removed and cells were washed three times with PBS (Life Technologies, Carlsbad, CA, USA) to remove virus particles. Cells were supplemented with fresh differentiation medium and kept in culture until indicated readout times.

### Treatments with recombinant αSyn

Cells were treated with 3 µM recombinant full-length and fragmented wild-type human αSyn (rPeptide, Watkinsville, GA) after 4 days of differentiation. Except for uptake experiments, treatments were kept on the cells for 48 h. Cells were then washed 3 times with PBS and treated with trypsin-EDTA (Life Technologies) for 45 s at 37 °C to remove extracellular αSyn, followed by a wash with DMEM/F12 medium supplemented with 10% fetal calf serum (FCS; Gibco Life Sciences, Carlsbad, CA), then a final wash in PBS. Cells were either harvested immediately after washing or supplemented with fresh differentiation medium and kept in culture until indicated readout times.

### Generation of αSyn knockout LUHMES cell line by CRISPR-Cas9 genome editing

To target all known splicing variants of the *SNCA* gene and to exclude the possibility of alternative translation initiation, we targeted *SNCA* exon 4. The 3’ portion of *SNCA* exon 4 and part of its adjacent intron were substituted with an autonomous puromycin resistance cassette on one allele and introducing a frame shift-inducing indel in the same exon on the second allele. The resistance cassette was designed for genome editing in human cells, harboring a human-codon-optimized *pac* gene ORF, driven by the human eEF1α promoter and terminated by bpA, interspaced by unique restriction sites for modular exchange of the elements situated in the pUC57 backbone (purEFlip_VE/pUC57). Small guide RNAs (sgRNAs) were designed using Benchling Biology Software 2016 (Benchling, San Francisco, CA, https://benchling.com) against *SNCA* exon 4 (5′-AGTAGCCCAGAAGACA GTGG-3′) and the adjacent 3′ intron (5′-GGAGCAAGATACTTACTGTG-3′). They were then cloned into the pbs-U6-chimaric_RNA sgRNA expression plasmid. A fragment of approximately 2 kb, harboring the exon, was amplified from LUHMES DNA via PCR and inserted into the pCR-Blunt-II vector (Invitrogen, Carlsbad, CA). The portion between guide RNA-binding positions was consecutively substituted by the puromycin selection cassette, disrupting their target recognition sequences and thus producing the final homologous donor vector. For expression of SpCas9 nuclease, the pCAG-Cas9v2-bpA vector was used. All plasmids were amplified in *E. coli* DH5-α and isolated using PureLink HiPure plasmid purification kits (Invitrogen, Carlsbad, CA, USA).

LUHMES cells were cultured in standard LUHMES growth medium at 37 °C/5% CO_2_. All vessels were pre-coated with 1% Geltrex (Gibco Life Sciences) in DMEM/F12 (Sigma-Aldrich, St. Louis, MO, USA) at 37 °C overnight. At 70% confluency, cells were washed once with PBS and then detached using Accutase (Sigma-Aldrich) for 15 min at 37 °C. Detached cells were washed in pre-warmed (37 °C) growth medium supplemented with 10% FCS, pelleted for 5 min at 270*g*, resuspended in growth medium and counted. Two million cells were then re-pelleted for 5 min at 100*g* and resuspended in 100 µL nucleofection solution (Amaxa Basic Nucleofector Kit Primary Neurons; Lonza, Basel, Switzerland) supplemented with the appropriate plasmids (10 µg for 10^6^ cells at a mass ratio of 2:1:1:1 [Cas9: homologous donor: exonic sgRNA: intronic sgRNA]). Cells were then immediately transferred into cuvettes and transfected using program C-013 on a Nucleofector 2b device (Lonza, Basel, Switzerland) according to the manufacturer’s protocol^18^. Finally, the cells were allowed to recover in 900 µL of pre-warmed RPMI medium with 20% B27 (Gibco Life Sciences) at 37 °C for 10 min and added to 5 mL of pre-warmed growth medium in a T25 flask. On the following day, cells were washed once with PBS and grown further in 5 mL of growth medium. After a first expansion of 2 to 3 days, transfected cells were re-plated in T25 flasks and supplemented with 0.2 µg/mL puromycin on the next day. After another 2 to 3 days, cells were washed with PBS and allowed to recover in growth medium for several days. The cells were then re-plated in 5 mL growth medium in T25 flasks and the medium supplemented with 0.8 µg/mL puromycin on the next day. After another 2 to 3 days, cells were washed and allowed to recover in growth medium. After one additional passage the cells were then seeded at 300 cells per 1.5 mL growth medium supplemented with 8% B27 and 10 µg/mL ciprofloxacin in 6-well plates and grown for a week at 37 °C, 5% CO_2_ and 3% O_2_. After clonal expansion, the clones were incubated in 0.02% EDTA/PBS (Sigma-Aldrich) for 4 min at 37 °C, PBS was added and individual cell patches were transferred by pipette into 300 µL pre-warmed growth medium supplemented with 6% B27 in 48-well plates. Individual clones were expanded and passaged, using a portion of the cell mass for genotyping. Selection criteria were integration of the resistance cassette into one allele and frame shift-inducing indels in the second allele of the *SNCA* gene. Absence of α-synuclein protein was confirmed by Western blot.

### Western blotting

Cells were harvested in M-PER lysis buffer (Thermo Scientific Pierce Protein Biology, Waltham, MA) supplemented with protease and phosphatase inhibitors cocktail (Roche, Basel, Switzerland). Lysis was carried out by 15 min incubation on ice, followed by one freeze-thaw cycle. Cell lysates were cleared by centrifugation at 13,000*g* for 10 min at 4 °C. Concentration of the supernatant was determined by BCA protein assay kit (Thermo Scientific Pierce Protein Biology) according to the manufacturer’s instructions.

CM samples were centrifuged at 2000*g* for 10 min to remove cell debris. Medium was then concentrated for Western blot analysis with a 3 kDa molecular weight cut-off filter (Vivaspin; Sartorius, Göttingen, Germany). The protein content in the medium was quantified using the 660 nm Protein Assay Reagent (Thermo Scientific Pierce Protein Biology).

Proteins from cell homogenates were loaded on 4–12% Bis-Tris precast protein gels (Bio-Rad Laboratories, Hercules, CA) and electrophoresis was carried out with MES running buffer. For CM samples, proteins were loaded on 16.5% Tris-Tricine precast protein gels (Bio-Rad Laboratories) and electrophoresis carried out with Tris-Tricine running buffer. Proteins were transferred on 0.2 µm PVDF membranes (Bio-Rad Laboratories) and immediately fixed with 0.4% formaldehyde as previously described^54^. Membranes were blocked with a 30% RotiBlock solution (Carl Roth, Karlsruhe, Germany) or 5% skimmed milk in Tris-buffered saline (TBS) supplemented with 0.05% Tween-20 (Sigma-Aldrich) (TBST) and incubated overnight with primary antibodies at 4 °C. Correspondent HRP-coupled secondary antibodies were incubated for 1 h at room temperature, followed by incubation in Clarity Western ECL Substrate (Bio-Rad Laboratories) for visualization. Images were taken with Odyssey Fc (LI-COR Biotechnology, Lincoln, NE) imaging system. The following primary antibodies were used: C-terminal antibody: rabbit anti αSyn (1:500; Cell Signaling Technology, Danvers, MA), N-terminal antibody: rabbit anti αSyn [EP1646Y] (1:500; Abcam, Cambridge, UK), rabbit anti GAPDH (1:3000; Merck Millipore, Billerica, MA), HRP coupled β-actin antibody (1:2000; Cell Signaling Technology). The following secondary antibody was used: anti-rabbit IgG (1:5000; Vector Laboratories, Burlingame, CA).

### LC/MS-MS analysis

Gel slices (2 mm) were excised from a Coomassie brilliant blue stained SDS-polyacrylamide gel at the regions between 5 and 18 kDa (fragments and monomer). Gel slices were further cut into small cubes and transferred into individual vials for subsequent protein cleavage by trypsin and chymotrypsin. First, gel pieces were destained with 50 mM ammonium bicarbonate (Ambic) (Sigma-Aldrich) in water containing 50% acetonitrile (ACN) (Sigma-Aldrich). Next, 200 ng of trypsin was added for the first digestion step and the samples were incubated for 12 h at 37 °C. The supernatant was transferred into a fresh vial and the gel cubes were washed once with 50 µL of a 100 mM Ambic solution and the wash was combined with the supernatant from the tryptic cleavage. Subsequently, 300 ng of chymotrypsin were added to the gel cubes and samples were incubated for 4 h at 30 °C. All samples were then acidified using 10% formic acid (FA) and corresponding supernatants of trypsin and chymotrypsin digestion were combined. Peptides were finally eluted twice with 50 µL of 50% ACN containing 0.1% FA (Sigma-Aldrich). All samples were vacuum-dried before desalting on a C18 stage tip column (Thermo Fisher Scientific). Desalted samples were then reconstituted in 0.05% trifluoroacetic acid (Sigma-Aldrich) and chromatographically separated on an U3000 nano-chromatography system (Thermo Fisher Scientific) which was directly coupled to an LTQ orbitrap mass spectrometer (Thermo Fisher Scientific) for online detection of peptides. Peptides were directly loaded on the RP-C18 nano-column packed into an ESI emitter (120 × 0.075 mm, filled with ReproSil-Pur C18-AQ 2.4 µm, Dr. Maisch AG, Ammerbuch-Entringen, Germany) and subsequently separated via a linear gradient from 3% ACN to 35% ACN in 50 min. Peptides eluting from the chromatography column were transferred into the mass spectrometer via nano-electrospray ionization. The mass spectrometer was run in data-dependent acquisition mode, acquiring one survey scan to detect ionized peptide ions and performing 6 fragment ion scans of selected precursor peptide for sequence determination.

### Proteomic data analysis

All raw data files for excised αSyn gel slices were searched in parallel against a human protein database (uniprot, vs 11/2017) using the MaxQuant/Andromeda search algorithm (vs. 1.6.0.6, www.maxquant.org^55^).

For comparison with calculated peptide masses, a mass deviation of 20 ppm was allowed for the first search step, for the main search it was adjusted to 6 ppm. For MS/MS data a mass accuracy for 0.6 Da was selected. Protein N-terminal acetylation, methionine oxidation and cysteine carbamidomethylation were enabled as post-translational modifications. All obtained results were filtered for 1% FDR on the peptide level and 5% on the protein level. Furthermore, at least two peptide sequences had to be associated with reported protein sequences.

For αSyn, N-terminal and C-terminal regions were not covered by the proteomics data. In order to guarantee correct annotation of the covered areas, identified peptides were loaded into Skyline (vs. 18, University of Seattle, www.skyline.ms^56^) and the peptide peak areas were annotated manually. Peptide abundance was compared between all samples.

### In vitro cleavage assay

Recombinant FL-αSyn (1 µg) was added to 100 µL of various unconcentrated media conditions to monitor proteolytic events. The mixture was incubated at 37 °C for 24 h. Next, 30 µL were taken out of the reaction, mixed with reducing XT sample loading buffer (Bio-Rad Laboratories) and heated at 95 °C for 5 min. Samples were loaded on in-house casted 16% Bis-tris gels prior to Western blotting.

### Conditioned medium uptake assay

Wild-type LUHMES were transduced with αSyn overexpressing AV and the resulting conditioned medium (CM) was harvested at DIV8. The medium was first spun at 2000*g* for 10 min to remove cell debris. CM was concentrated using 3 kDa molecular weight cut-off filters (Vivaspin; Sartorius, Göttingen, Germany) to achieve the desired concentration factors (2X, 5X and 10X) accordingly. DIV8 αSyn knockout LUHMES cells were treated with different concentrations of the conditioned medium at 37 °C for 6 h. Cells were afterwards thoroughly washed, harvested and cell lysates were used for Western blotting.

### Recombinant protein labeling and uptake assays

For protein uptake, recombinant proteins were labeled with ATTO-488-NHS ester fluorescent dye (ATTO-TEC, Siegen, Germany) according to the manufacturer’s instructions. Briefly, protein concentrations were adjusted to 2 mg/mL and reconstitution buffer was exchanged from Tris to PBS with dialysis. The pH of the coupling reactions was adjusted to 8.3 with a 0.2 M sodium bicarbonate solution (Sigma-Aldrich). The fluorescent dye was reconstituted with dimethyl sulfoxide (Sigma-Aldrich) to a final concentration of 5 mg/mL and added to the proteins with a three to ten folds molar excess. The reaction was carried out at room temperature for 1 h in the dark. The excess unbound dye was removed with Bio-Spin 6 size exclusion spin columns (Bio-Rad Laboratories). Labeling was verified by running the labeled proteins on a gel followed by visualization with a fluorescent filter. For confocal imaging, cells were plated on 8-well ibidi µ-slides (ibidi, Gräfelfing, Germany). After four days of differentiation, cells were treated with 3 µM of FL-αSyn or fragments for 48 h. The treatment was removed and cells were washed as described above. CellTrace Calcein red-orange (Thermo Fisher Scientific) was used as a cell filling dye prior to fixing with 4% paraformaldehyde (Sigma-Aldrich). 4′,6-diamidino-2-phenylindole (DAPI; Invitrogen) was used as a nuclear counterstain. Z-stack images were taken using an inverted laser scanning confocal microscope (Zeiss LSM 880, Carl Zeiss, Oberkochen, Germany) using a 40x oil immersion objective with a digital zoom of 2.5. Orthogonal projections were made using the Fiji software (https://imagej.net/fiji).

For automated uptake kinetics assays, cells were seeded on 48 well-plates and treated with labeled αSyn as described above. At the indicated time points, cells were washed twice with PBS and then treated with a trypan blue solution to quench extracellular green fluorescence as previously described^57^. Intracellular fluorescence in live cells at each time point was assessed with a CLARIOstar plate reader (BMG Labtech, Offenburg, Germany) using a well-scanning protocol with a 10 × 10 matrix.

### LDH assay

Cell death was quantified by measurement of lactate dehydrogenase (LDH) released into the culture medium at the indicated readout times using the fluorescence based CytotoxONE Membrane Integrity Assay (Promega, Fitchburg, WI) according to the manufacturer’s instructions. Fluorescence levels were determined with a fluorescence microplate reader CLARIOstar plate reader (BMG Labtech). Cells lyzed with 1% triton X (Sigma-Aldrich) were used as a positive control and were considered as the 100% cell death reference.

### Aggregation assays

The aggregation reactions were prepared as follows: the different fragments that were used as seeds were mixed with monomeric FL-αSyn with a molar ratio of 1:10, respectively, with a final concentration of 20 µM in 50 mM tris-HCl buffer at pH 7.0 and 25 µM thioflavine T (ThT; Sigma-Aldrich). Each sample was run in triplicates in black-walled clear bottom 384 well plates (Thermo Fisher Scientific). The final volume of the reactions was 45 µL. The plates were sealed and allowed to shake at 700 rpm at 37 °C for 230 h. Aggregation kinetics were determined by measuring ThT fluorescent signal every hour in relative fluorescence units (RFU). The experiment was repeated at least 3 times.

In order to determine the apparent lag time of the different reactions, each data set was normalized by subtraction of its lowest value and division by its highest value. Apparent lag times were determined as the time at which the curves reached 10% of the elongation phase (T_10%_). Growth rate was calculated as follows: Growth rate (RFU/hour) = (*F*_50%_ − *F*_10%_)/(*T*_50%_ − *T*_10%_), where *F*_50%_ and *F*_10%_ are the fluorescence values in RFU when the curves reached 50 and 10% of the elongation phase, respectively. *T*_50%_ and *T*_10%_ are the time points at which the curves reached 50 and 10% of the elongation phase, respectively.

### Dynamic light scattering measurements

Particle size distributions and zeta potential measurements were obtained with DLS using a Malvern Zetasizer Nano ZSP (Malver Instruments, Malvern, UK). To obtain an estimation of the hydrodynamic radius (*r*_h_), the Stokes-Einstein relation was used:

$$D = \frac{{k_BT}}{{6\pi \eta r_h}}$$

where *D* is the measured diffusion constant of the particle, *k*_B_ the Bolzmann constant, *T* absolute temperature and the viscosity of the medium. The particle diameter (2xr_h_) was calculated from the Z-average size from the cumulants fit, using the software provided by Malvern Instruments.

### Proteinase K digestion

For recombinant fibrils produced as a 1:10 mixture between fragments and FL-αSyn, 3 µg of fibrils were digested at 37 °C with 0.1 µg/mL of proteinase K (Thermo Fisher Scientific, Waltham, MA, USA) for the indicated amounts of time. The digestion reaction was stopped by mixing in preheated sample loading buffer followed by immediate incubation at 95 °C for 5 min. The samples were then loaded on 12% Bis-Tris criterion gels (Bio-Rad Laboratories). After electrophoresis, the gels were stained with Pierce silver staining kit (Thermo Fisher Scientific) according to the manufacturer’s instructions.

For cell lysates, 20 µg of total cell lysates were digested at 37 °C with increasing concentrations of proteinase K for 30 min. The digestion reaction was stopped by mixing in preheated sample loading buffer followed by immediate incubation at 95 °C for 5 min. The samples were then loaded on 12% Bis-Tris criterion gels (Bio-Rad Laboratories) and Western blots were carried out as described above.

### Statistical analysis

Biological repeats were defined as independent experiments and technical repeats were defined as repeats within the same biological repeat. Each experiment includes at least 3 independent repeats. Statistical analysis was performed using GraphPad Prism 7.01 (GraphPad Software, La Jolla, CA). The identify outliers test was performed with GraphPad Prism using the ROUT method (Q = 1%) and outliers were excluded from statistical analysis. Data are shown as means with error bars representing the standard error of the mean (SEM). Two-way ANOVA and one-way ANOVA were used followed by Tukey’s post hoc test, *p*-values < 0.05 were considered as statistically significant.

## Supplementary information

Figure S1

Figure S2

Figure S3

Supplementary figure legends
