# Supplementary figures and images for: Alpha-synuclein fragments trigger distinct aggregation pathways
Source: Cell Death Dis. 2020 Feb 3;11(2):84. doi: 10.1038/s41419-020-2285-7 (PMC6997403; doi:10.1038/s41419-020-2285-7)

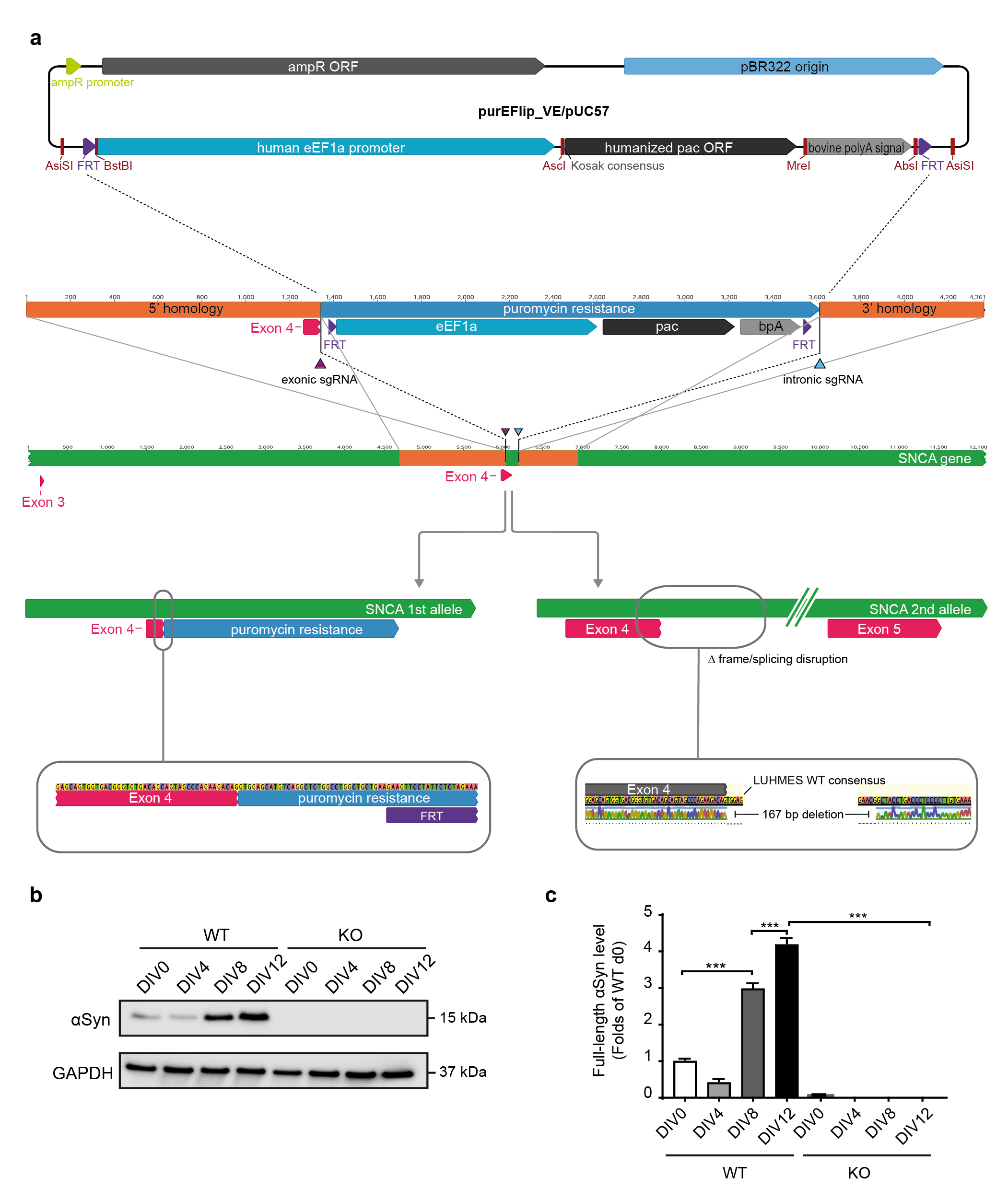

Supplement: Supplementary file 1 — Figure S1 [file 41419_2020_2285_MOESM1_ESM.png]

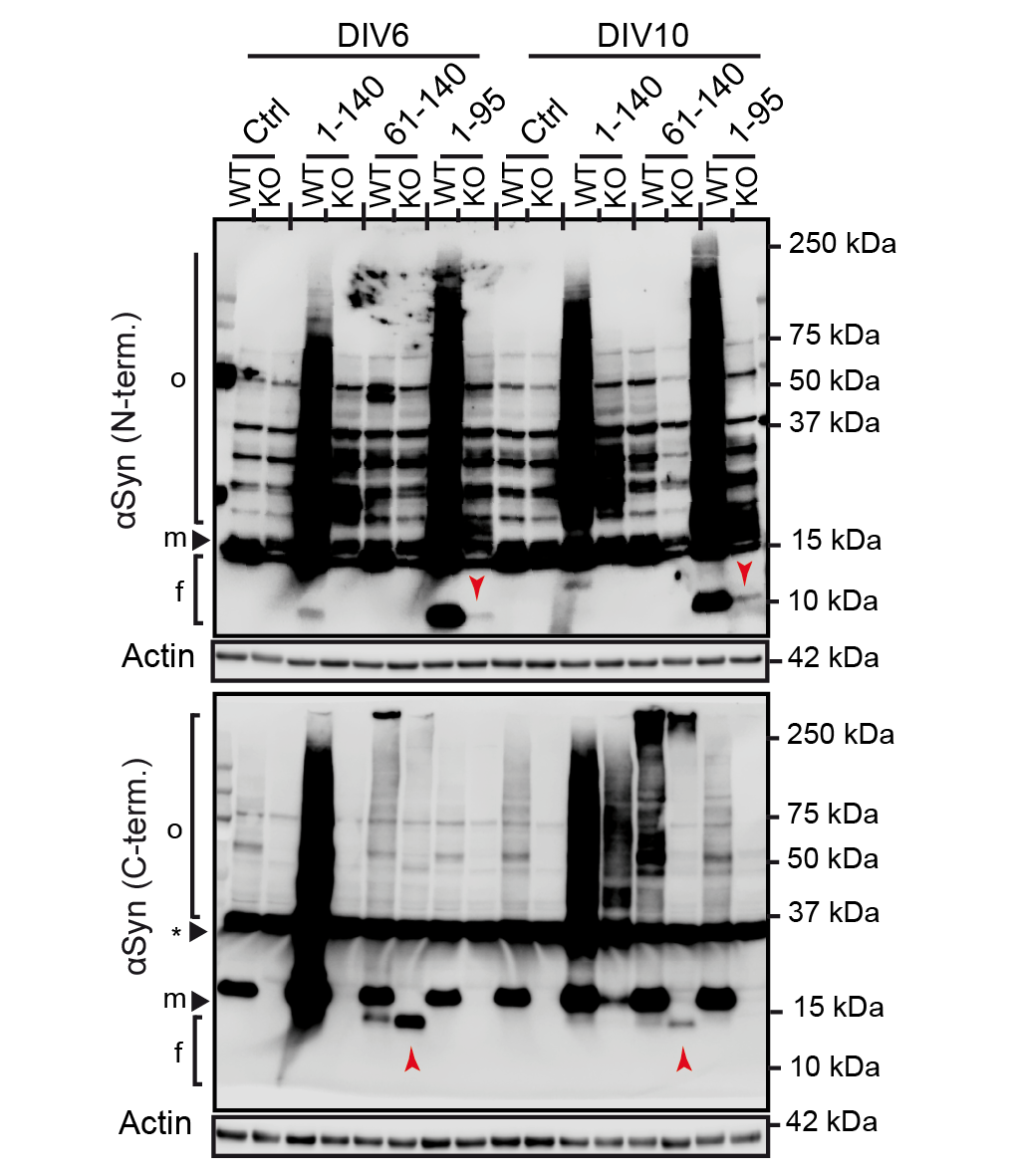

Supplement: Supplementary file 2 — Figure S2 [file 41419_2020_2285_MOESM2_ESM.png]

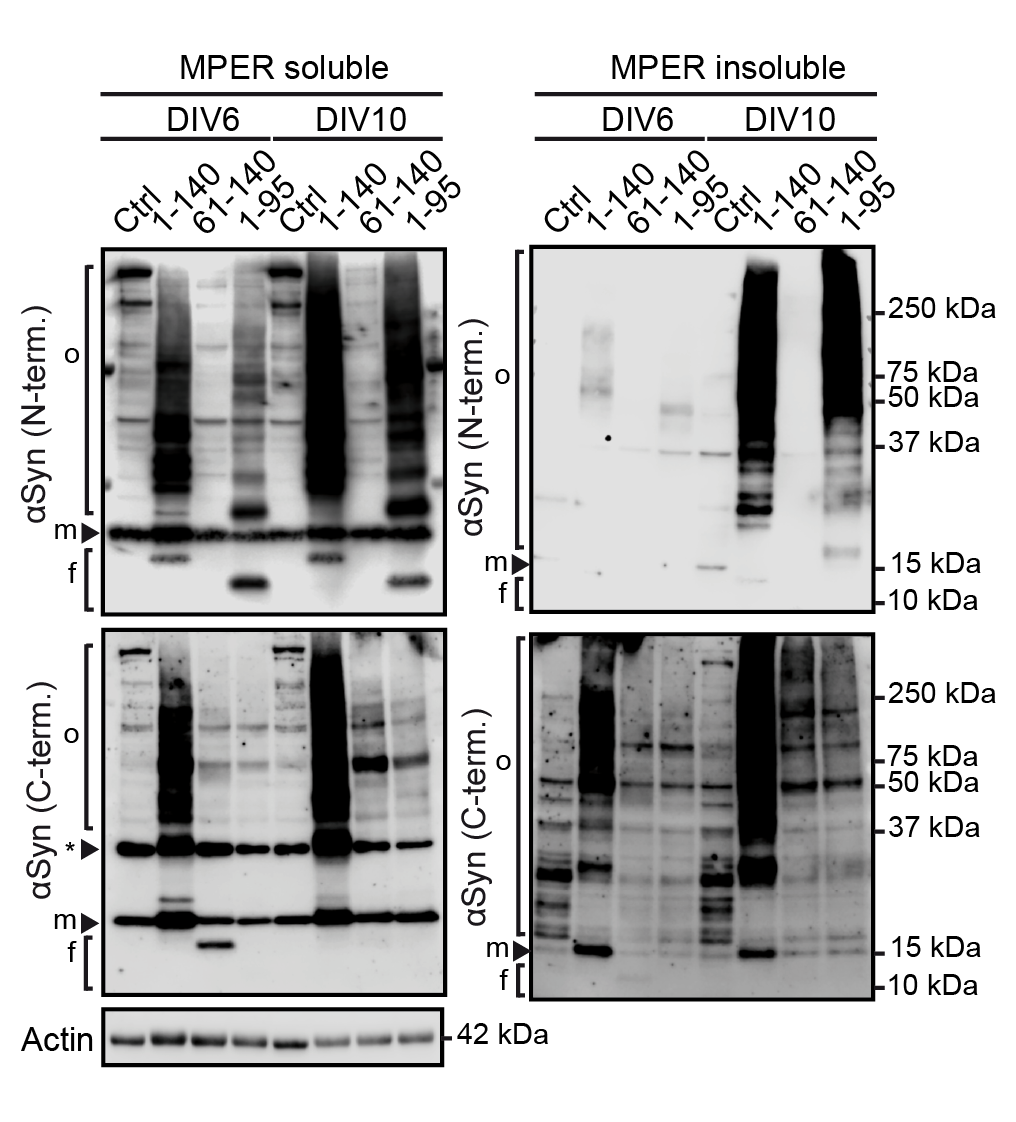

Supplement: Supplementary file 3 — Figure S3 [file 41419_2020_2285_MOESM3_ESM.png]
