# Supplementary material for: Alpha-synuclein fragments trigger distinct aggregation pathways
Source: Cell Death Dis. 2020 Feb 3;11(2):84. doi: 10.1038/s41419-020-2285-7 (PMC6997403; doi:10.1038/s41419-020-2285-7)
Supplement: Supplementary file 4 — Supplementary figure legends [file 41419_2020_2285_MOESM4_ESM.docx]

**Supplementary information**

**Alpha-synuclein fragments trigger distinct aggregation pathways**

**Tasnim Chakroun, Valentin Evsyukov, Niko-Petteri Nykänen, Matthias Höllerhage, Andreas Schmidt, Frits Kamp, Viktoria C. Ruf, Wolfgang Wurst, Thomas W. Rösler, and Günter U. Höglinger**

**Contents**

**Supplementary figure legends**

**Figure S1 . Generation and Validation of the αSyn Knockout (KO) LUHMES Cell Line**

**(a)** Genomic engineering strategy of the LUHMES *SNCA* locus at exon 4. A knock-in vector (backbone: pUC57) harboring an autonomous human-optimized puromycin resistance cassette, flanked by a total of ~ 2 kb sequence homology to the LUHMES *SNCA* locus around exon 4, was used to replace portions of exon 4 and the adjacent 3’ intron on one allele via the CRISPR/Cas9 system. Simultaneously, the second *SNCA* allele was disrupted during the genome editing process with the same specific single guide RNAs (sgRNAs) used for the knock-in. The knock-in on the first allele results in frame-disruption and precipitates premature termination of transcription, whereas the deletion on the second allele leads to early translation termination and ablation of the splicing boundary at the exon 4/intron 4 juncture. Grey boxes represent zoom panels to depict the exact genomic structure of both alleles. FRT: flippase recognition targets, bpA: bovine poly-A signal, eEF1a: promoter region of human eukaryotic elongation factor 1-α 1, ORF: open reading frame, pac: puromycin N-acetyltransferase. Dark red: restriction endonuclease sites for modular cloning.

**(b-c)** Validation of CRISPR/Cas9 αSyn knockout (KO) LUHMES cells. Wild type (WT) and αSyn KO LUHMES cells were differentiated for the indicated amount of time prior to harvest. The αSyn protein levels at different developmental stages were assessed by Western blot. (**b**) shows a Western blot of αSyn in WT and KO LUHMES cells at the indicated times. While WT cells strongly upregulate αSyn during differentiation, no αSyn can be detected in the knockout cells even after prolonged culture. GAPDH was used as loading control. (**c**) shows a quantification of αSyn monomer levels normalized with GAPDH. Data are presented as mean + SEM from 3 biological repeats. *** p < 0.001; one-way ANOVA with Tukey’s *post hoc* test.

**Figure S2. Overexposed Western Blots of Intracellular Seeding Potency of Recombinant Full-length αSyn and Fragments in Wild-type (WT) and αSyn Knockout (KO) LUHMES neurons**

Overexposure of the Western blot membrane allows to visualize αSyn fragments 1-95 (upper panel) and 61-140 (lower panel) in αSyn KO cells (red arrowheads).

**Figure S3. Soluble and Insoluble Intracellular Aggregation Seeded with Recombinant Full-length αSyn and Fragments**

Seeding potency of recombinant full-length and fragmented αSyn in MPER-soluble and MPER-insoluble fractions of cell homogenates. Cells were treated with recombinant αSyn for 48h and harvested at DIV6 and DIV10. 1% sarkosyl was used to solubilize the MPER insoluble material. Western blots with N-term. and C-term. antibodies reveal an accumulation of insoluble αSyn from DIV6 to DIV10. f: fragments, m: monomer, o: oligomer, *: unspecific band. Actin was used as loading control.
